# Radiation-induced rectovaginal fistulas in locally advanced gynaecological malignancies—new patients, old problem?

**DOI:** 10.1007/s00423-016-1539-4

**Published:** 2016-12-16

**Authors:** Piotr Zelga, Marcin Tchórzewski, Marta Zelga, Janusz Sobotkowski, Adam Dziki

**Affiliations:** 1 Department of General and Colorectal Surgery, Medical University of Lodz, Plac Hallera 1, 90-647 Lodz, Poland; 2 Brachytherapy Unit, Regional Oncological Centre in Lodz, Pabianicka 62, Lodz, Poland

**Keywords:** Rectovaginal fistula, Diverting stoma, Radiation therapy

## Abstract

**Purpose:**

Radiation-induced rectovaginal fistula (RI-RVF) is a chronic and serious condition with a significant influence on quality of life. The aim of this study is to evaluate the results of surgical treatment of rectovaginal fistulas of patients previously undergoing radiotherapy.

**Methods:**

Fifty patients treated in the Gynaecological Radiotherapy Unit for gynaecologic malignancy and in the Department of General and Colorectal Surgery for RI-RVF between 2003 and 2013 were enrolled into a prospectively maintained database and underwent regular follow-up examinations in an outpatient clinic, during which surgical outcomes were assessed.

**Results:**

Median age was 60 years (range 40–84 years). Cervical cancer was the most common cause of radiotherapy. Median time of fistula development after radiotherapy was 20 months (range 5–240 months). In 48 (96%) patients, only faecal diversion could be performed, while two patients underwent rectal resection. The fistula healed in six patients. Factors that correlated with fistula healing were a distance from the anal verge above 7 cm (*p* = 0.007 OR 18 95%CI 2.2609–14.3062) and creation of loop ileostomy (*p* = 0.08 OR 17 95%CI 1.2818–23.9701), whereas a prolonged course of radiotherapy of more than 6 weeks (*p* = 0.047) correlated negatively. In multivariate analysis, only distance from the anal verge remained significant (*p* = 0.031 OR 2.35 95%CI 1.0422–5.2924).

**Conclusions:**

The treatment of radiation-induced rectovaginal fistulas needs to be tailored individually to each patient. Faecal diversion remains the simplest and safest method of treating RI-RVF, especially in the group of patients who cannot undergo complicated surgical procedures, and offers acceptable quality of life.

## Introduction

Rectovaginal fistula is a serious anorectal condition with a significant impact on patient quality of life. In recent years, new options of surgical treatment have been introduced or rediscovered, enabling improved treatment results, especially when dealing with low fistulas [1, 2]. However, such progress has not been observed in the field of Crohn-related and radiation-induced RVF (RI-RVF), where the inflammatory response and fibrosis of surrounding tissue limits the possibilities of tissue healing, thus decreasing the number of possible interventions [3–5]. Despite promising reports concerning the use of collagen plugs and the Martius flap, insufficient studies exist to properly assess the value of proposed methods, especially in the conditions mentioned below [6, 7]. Moreover, the majority of RI-RVF patients are older, often with significant comorbidities and decreased capability of applying postoperative recommendations, which also negatively influences the outcomes. Therefore, diverting colostomy is often the only possible treatment, allowing palliation of symptoms, a reduction of regional sepsis and improved tissue healing. Although spontaneous closure of RI-RVF is rarely seen after faecal diversion alone, the need for better local control of the disease warrants further attempts to repair RI-RVF with curative intent [8].

The overall success rates for RI-RVF treatment vary from 18 to 93%, depending on the methods applied [4, 9–11]. However, drawing wider conclusions is limited, since the majority of the reported studies are based on analyses of relatively small numbers of patients, often not exceeding 20 [12–14].

The present study assesses the results of RI-RVF treatment and analyses the potential predictive factors of treatment outcomes based on the experience of a single centre over a 10-year period.

## Materials and methods

### Patient recruitment

The Department of General and Colorectal Surgery, Medical University of Lodz, Poland, is a regional tertiary referral centre and a national centre of excellence for colorectal diseases, serving a population of about 3 million people. In 2003, after institutional review board approval, the Rectovaginal Fistula database was established to prospectively gather demographic and clinical data based on established protocols.

### Inclusion criteria and process

Each patient referred to the department with a diagnosis of radiation-induced rectovaginal fistula was carefully examined by a team of two colorectal surgeons (junior and senior) to confirm the presence of RI-RVF and classify it, based on diameter and distance from the anal margins, using a rectoscope. RI-RVF was defined as the presence of an abnormal canal between the rectum and the vagina, causing involuntary loss of faecal material and gas and intermittent discharge of mucus through the vagina, which developed no earlier than 90 days after completion of radiotherapy, after the exclusion of other underlying ethiologies (i.e. IBD, cancer, iatrogenic). The diameter of the fistula was defined as the distance between two points located on the extremities of the fistula opening in the rectum along its longer axis measured during rectoscopy or in computed tomography studies. Fistulas with a diameter smaller than 0.5 cm were considered as small, between 0.5–2.5 cm as medium sized and larger than 2.5 cm as large.

To eliminate any possible bias in comparing different treatment schemes, only patients with cervical and endometrial cancer were included in the study. On the basis of a bilateral agreement between Department and Gynaecological Radiotherapy Unit, data concerning the course of oncologic treatment for a particular patient was transferred to the database and linked with medical records concerning gynaecological treatment of the primary disease. Medical data unrelated to the main diagnosis (comorbidities and remaining medical history) was gathered from the patients based on discharge letters. On this basis, the patient was qualified for surgery. Positive treatment outcome was defined by the absence of any vaginal discharge of faeces, flatus or mucous and the absence of any visible fistula tract identified by computer tomography (CT) of the abdomen with rectal contrast. Persistence of clinical symptoms of rectovaginal fistula and/or presence of fistula, confirmed by radiological studies (CT with rectal contrast) after the operation was considered as a negative outcome.

The exclusion criteria comprised the presence of RVF with an aetiology which was not radiation induced (e.g. Crohn disease), patients with active cancer or recurrence, and incomplete medical records.

### Primary disease treatment

In order to compare administered radiation doses, for high-dose rate (HDR) and low-dose rate (LDR), fractional doses were presented as equivalent doses in 2 Gy fractions (EQD2). Patients with cervical and endometrial cancer were treated by intracavitary brachytherapy (ICBT) as HDR to the 35 Gy/5 fraction (EQD2 39.70 Gy) or LDR to the 54 Gy/3 fraction (EQD2 58Gy) and external beam radiotherapy (EBRT) to the 22 fraction of 2 to 44 Gy. Additionally, four doses of cisplatin 50 mg/m^2^ were added during EBRT. The cumulative radiation dose to point A was around 84 Gy for no longer than 8 weeks. The adjuvant radiotherapy was routinely administered also in cancer stage I patients with high-risk factors including cancer histological type (other than adenocarcinoma), grade (grade 3), depth of myometrial invasion (stage IB) and lymphovascular invasion (less than 10 nodes assessed). Complications after radiotherapy were classified and graded according to the Radiation Therapy Oncology Group scale [15]. Acute complications were defined as developing during treatment or within 90 days after its completion. Late complications were those occurring more than 90 days after the end of treatment. Only grade III–IV complications were included in the analysis. RI-RVF was not included in the grade 4 late complication group, as this group was reserved for the complications that coincide with RI-RVF. Tumours in stages IB1 to IIA were initially treated with radical panhysterectomy and removal of the lymph nodes.

### Follow-up

Each patient was scheduled for follow-up visits in the Departmental Outpatient Clinic (DOC) after 1 week and then 1 month following discharge from hospital, and once a year after that. Early surgical complications were defined as any deviation from the normal postoperative course occurring within 30 days following the operation, while late complications were those occurring after this period. When the presence of late complications was suspected, further visits were organized. In the case of patients with disabilities or referrals from distant regions, regular structured phone interviews were performed in the periods stated above and results of the physical and radiological examinations performed in the health institution local to the patient were sent to the department. In 2009 and in the end of the observation period (2015), all living patients who remained in follow-up were examined in an outpatient clinic by surgeons involved with the study.

### Quality of life

As a part of an ongoing study concerning the quality of life of patients with radiation proctopathy, all patients that were examined in 2015 were asked to participate also in that study and complete two questionnaires, i.e. standardized Satisfaction with Life Scale (SWLS) and standardized Acceptance of Illness Scale (AIS), adapted to Polish conditions by Juczynski [16]. Only the key findings concerning the obtained scores of the patients with RI-RVF are discussed in the present study.

### Statistical analysis

The collected data was entered by an encoder and checked for integrity by another encoder. The data files were compared and cross-checked for accuracy with the data collection forms after every ten patients. The data was analysed using STATISTICA (v.11 StatSoft, Inc., Tulsa OK, USA). Groups were compared using either the t test, for normally-distributed data, or the Mann-Whitney U test. Categorical variables were analysed using the chi-squared or Fisher’s exact test with the level of significance being *P* = 0.05. Frequencies (%) were calculated to describe patient characteristics and treatment outcomes.

## Results

### Patient characteristics

Fifty women were enrolled into the study. Demographic data and data concerning the primary disease are shown in Table 1. The median age of patients was 60 years (range 40–84 years) and the majority of patients lived in urban areas.

**Table 1:** Background data for patients with radiation-induced rectovaginal fistula

	Persistent	Healed	*p* value	Overall
Age (range)	60 (40–84)	63 (45–72)	0.8875	60 (40–84)
BMI (range)	24 (15.6–33)	23 (21–31.3)	0.714	24 (15.6–33)
Smoking status				
Current	15 (41.7%)	3 (50%)	0.261	20 (40.0%)
Former	21 (58.3%)	3 (50%)		30 (62.0%)
Blood count values on admission				
Hg (g/dl)	11.3 (8.6–14.8)	12.35 (10.4–12.2)	0.233	11.9 (7–14.8)
WBC (million cells/mcL)	9.09 (5.1–20.3)	6.61 (5.5–16.7)	0.242	9.37 (2.8–20.33)
ASA score				
I–II	20 (55.6%)	5 (83.3%)	0.373	30 (60.0%)
III–IV	16 (44.4%)	1 (16.7%)		20 (40.0%)
Comorbidities (Charlson score)				
0–2	23 (63.9%)	5 (83.3%)	0.645	35 (70.0%)
3–5	13 (36.1%)	1 (16.7%)		15 (30.0%)
Primary disease				
Cervical cancer	25 (69.4%)	4 (66.7%)	0.615	37 (74.0%)
Endometrial cancer	11 (30.6%)	2 (33.3%)		13 (26.0%)
Staging (FIGO scale)				
Ia	2 (5.6%)	0 (0%)	0.731	3 (6%)
Ib	6 (16.7%)	1 (16.7%)	0.690	9 (18%)
IIa	10 (27.8%)	2 (33.3%)	0.560	14 (28%)
IIb	8 (22.2%)	3 (50%)	0.313	12 (24%)
IIIa	3 (8.3%)	0 (0%)	0.621	3 (6%)
IIIb	7 (19.4%)	0 (0%)	0.566	10 (20%)
IVa	0 (0%))	0 (0%)	-----	0 (0%)
IVb	0 (0%))	0 (0%)	-----	0 (0%)
Histopathology				
Adenocarcinoma	10 (27.8%)	2 (33.3%)	0.561	12 (24.0%)
Squamous cell	25 (69.4%)	4 (66.7%)	0.615	36 (74.0%)
Undifferentiated carcinoma	1 (2.8%)	0 (0.0%)	0.857	1 (2.0%)
Treatment				
Surgery	21 (58.3%)	3 (50.0%)	0.519	27 (54.0%)
Chemotherapy	17 (47.2%)	4 (66.7%)	0.670	21 (42.0%)
Brachy and teletherapy	30 (83.3%)	4 (66.7%)	0.576	42 (84.0%)
Prolonged radiotherapy (more than 6 weeks)	18 (50.0%)	0 (0.0%)	**0.029**	18 (36.0%)
Reduced radiotherapy (less than 6 weeks)	5 (13.9%)	1 (16.7%)	0.629	6 (12.0%)
Only teletherapy	1 (2.8%)	0 (0.0%)	0.857	1 (2.0%)
Only brachytherapy	5(13.9%)	2 (33.3%)	0.567	7 (14.0%)
Early complications after radiotherapy grades III–IV	10(27.8%)	0 (0.0%)	0.308	10 (20.0%)
Late complications after radiotherapy grades III–IV	7 (19.4%)	1 (16.7%)	0.681	8 (16.0%)
RI-RVF treatment				
Loop ileostomy	1 (2.8%)	2 (33.3%)	**0.048**	9 (18.0%)
End ileostomy	0 (0.0%)	0 (0.0%)	–	1 (2.0%)
Loop transversostomy	21 (58.3%)	3 (50.0%)	0.519	24 (48.0%)
Loop sigmoideostomy	12 (33.3%)	1 (16.7%)	0.647	14 (28.0%)
Lower or Anterior resection	2 (5.6%)	0 (0.0%)	0.732	2 (4.0%)

Comorbidities were classified according to Charlson score

*RI-RVF* radiation-induced rectovaginal fistula, *FIGO* International Federation of Gynaecology and Obstetrics, *BMI* body mass index, ASA American Society of Anaesthesiologists

### Primary disease treatment

Twenty-seven patients (54%) underwent Wertheims-Meggs panhysterectomy before radiation treatment. Twenty-three patients received radiotherapy within standard time frames and doses. The median total dose of EBRT was 44 Gy (range: 31.4–52 Gy). The median dose per fraction was 2.0 Gy (range 1.8–2.0 Gy). The median total dose for HDR was EQD2 37.9 Gy (range 10.8–70 Gy) and for LDR was EQD2 58 (range 20.4–59.1 Gy). The median overall treatment time for radiotherapy was 49 days (range 14–140 days). The most frequent chemotherapy was concurrent delivery of cisplatin (14 patients) or Taxol (7 patients).

Symptoms of grade 3 or 4 acute treatment-related toxicity, as defined by the WHO, occurred in ten (17%) patients, including anaemia in five patients, nausea and vomiting in three, cystitis in three, and vomiting and diarrhoea in another three. Eight (14%) patients demonstrated late treatment complications of grade 3 or 4 toxicity other than RI-RVF: radiation enteritis, radiation cystitis, bowel obstruction, ileo-ileo fistula and recto-vesical fistula.

### Fistula characteristic

**Fig. 1:**
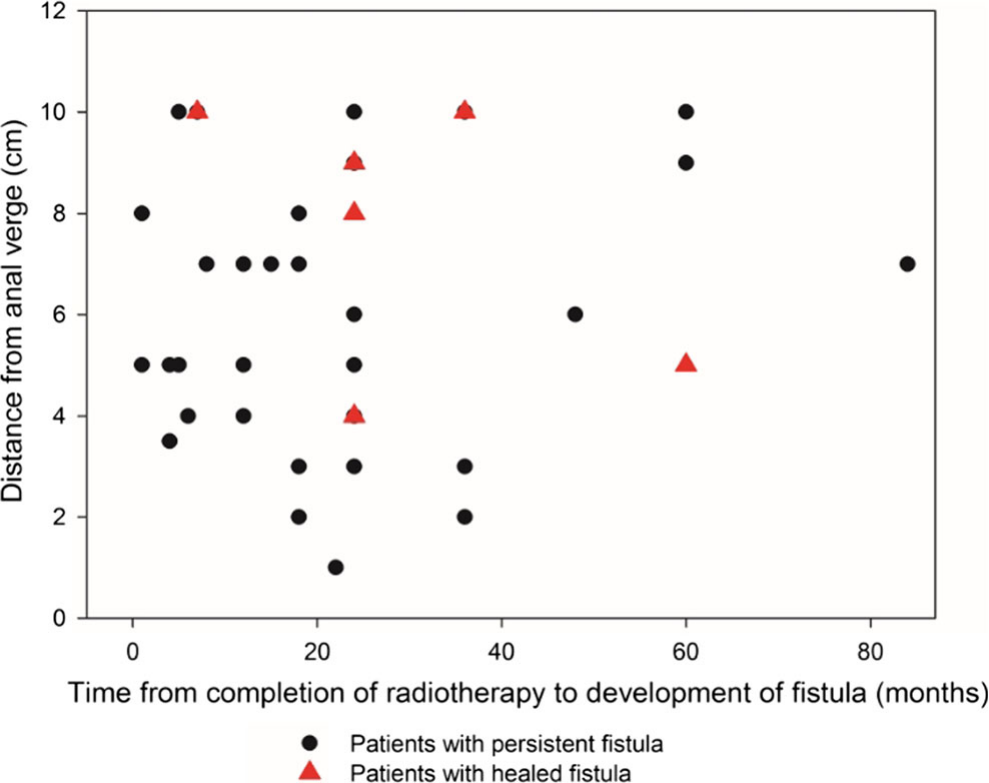
Time from radiotherapy and location of RI-RVF in patients with and without healed RI-RVF. Patients with healed RI-RVF are marked. A marked difference was found in distance from anal verge between patients with healed RI-RVF and patients with persistent RI-RVF (*p* = 0.008 95% OR 26.67 Cl 2.18–32.47)

Median time from radiotherapy to fistula development was 20 months (range 5–240 months). The majority of fistulas developed between 5 and 40 months, regardless of their location (Fig. 1).

Median duration of symptoms was 1.5 months (range 0.2–23 months). While 47 patients (94%) complained of passing a stool through the vagina, the remaining 3 reported a history of perianal pain, bloody discharges from the rectum and vagina and vaginal infection.

In total, 13 fistulas were classified as low, 33 as medium and 11 as high. In 12 patients, the opening of the fistula in the rectum could not be located during rectoscopy: the canal was further than 9 cm above the anal verge in 7 patients, and the opening of the fistula was smaller than 0.5 cm and could not be distinguished from the surrounding inflammatory tissue in 5. In ten cases, the fistula was identified during radiographic studies and in two cases by insertion of a tampon into the vagina followed by instillation of a methylene blue enema. While fistula diameter was less than 2.5 cm in 48 patients (80%), the opening in the rectum was wider in the remaining cases, up to even 3.5 cm.

RI-RVF coexisted with a vesicovaginal fistula in three patients and with an intestinovaginal fistula in another three. In another two patients, a vesicovaginal fistula developed in a later postoperative course.

### Treatment outcomes

**Fig. 2:**
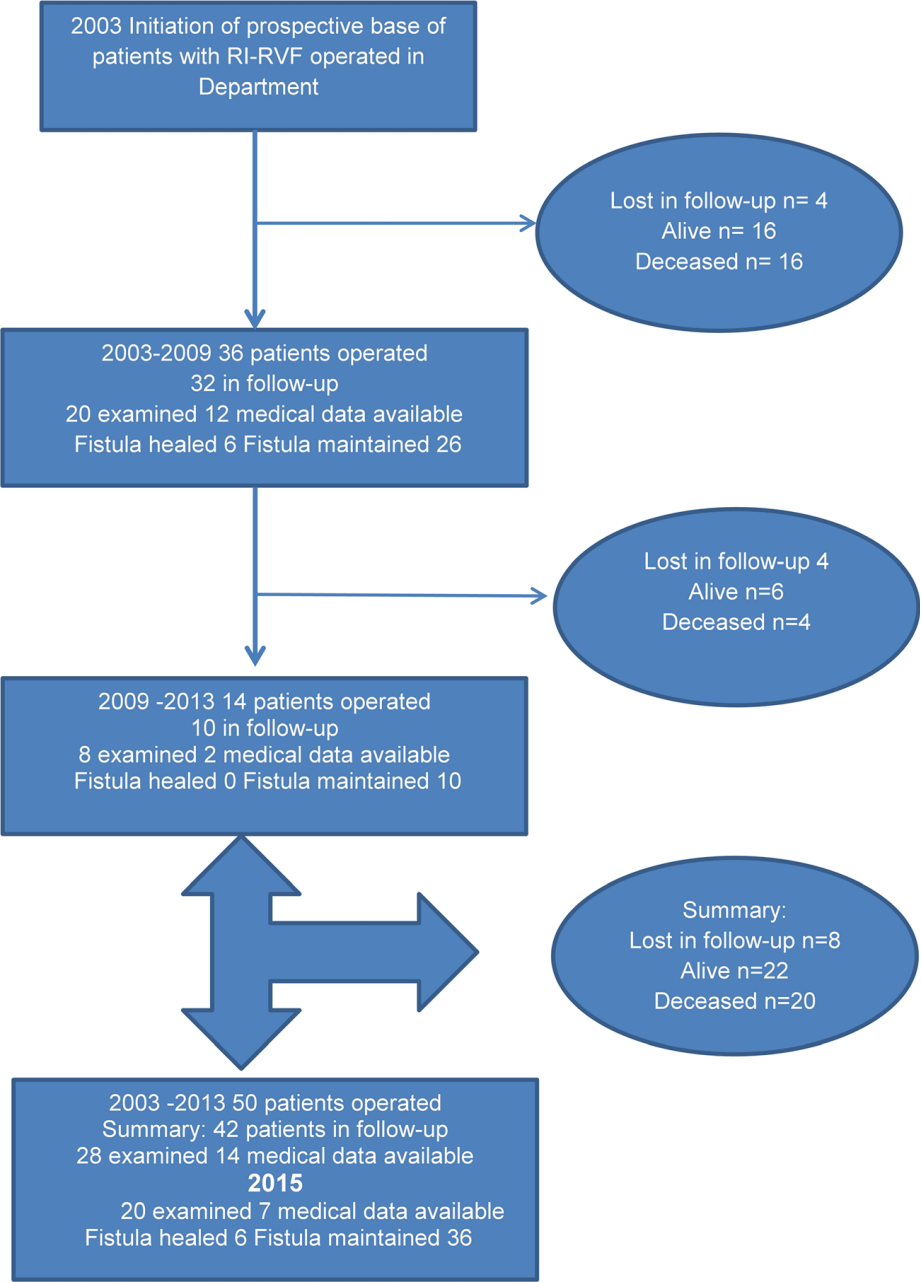
Follow-up scheme

By the end of the study, 22 patients were under observation and 20 patients had died. In summary, eight patients were out of follow-up and could not be contacted. The course of observation is presented in Fig. 2. Among the living patients, 20 remained in follow-up in the outpatient clinic and another 2 were available for examination when prompted. Median follow up in DOC was 2 years, whereas observation time was 3 years (range 1–15 years).

Faecal diversion alone was instigated in 97% of the patients. Two patients underwent lower anterior resection procedure (LAR), the former with primary anastomosis and the latter without.

The fistula healed spontaneously in six patients, with one of whom underwent restoration of the digestive tract. In the remaining five patients, the stoma was not reversed because of high perioperative risk or the decision of the patient to not undergo another operation. A summary of the patients is given in Table 2.

**Table 2:** Characteristics of six patients with healed radiation-induced rectovaginal fistulas

Number	Date of radiotherapy	Age of patient at diagnosis (years)	Surgery type	Histopathology	FIGO	Radiotherapy		Time from the end of radiotherapy to fistula formation (months)	Distance of the fistula from the anal verge (cm)	Fistula size (cm)	Operation performed	Time to fistula healing (years)
						**Teleradiotherapy**	**Brachytherapy**					
**1**	2001	66	**–**	Cervical squamous cell carcinoma	IIa	44 Gy	HDR EQD2 49.6Gy	60	5	≤0.5	Loop transversostomy	2
**2**	2001	47	Wartheim’s panhysterectomy	Endocervical adenocarcinoma	Ib	44 Gy	HDR EQD2 37.9Gy	24	8	1 (0.5–2)	Loop transversostomy with simple suturing of fistula. 1 year later LAR	1
**3**	2003	45	Wartheim’s panhysterectomy	Cervical squamous cell carcinoma	IIb	44 Gy	LDR EQD2 38.5Gy	24	4	≤0.5	Loop sigmoideostomy	2.5
**4**	2003	48	**–**	Cervical squamous cell carcinoma	IIa	44 Gy	HDR EQD2 37.9Gy	36	10	≤0.5	Loop ileostomy	2.5
**5**	2009	51	Wartheim’s panhysterectomy	Endocervical adenocarcinoma	IIb	44 Gy	HDR EQD2 37.9Gy	7	10	≤0.5	Loop ileostomy	2
**6**	2010	63	**–**	Cervical squamous cell carcinoma	IIb	44 Gy	HDR EQD2 42.7Gy	24	9	≤0.5	Loop transversostomy	1

*HDR* high dose radiotherapy, *LDR* low dose radiotherapy, *EQD2* equivalent doses in 2-Gy fractions

### Complications

Overall complications occurred in 43% of cases (25 patients): 22% (*n* = 13) being early and 28% (*n* = 16) late. One in-hospital death occurred. A summary of the complications is described in Table 3. Wound infection was the most commonly observed early complication (10%) and stomal prolapse the most common late complication (14%).

**Table 3:** Complications in patients with radiation-induced rectovaginal fistulas

Type of complication	Patients with healed rectovaginal fistula	Patients with persistent rectovaginal fistula	Overall	*p* value
Early	2 (33.3%)	9 (25.0%)	11 (22.0%)	0.503
Wound infection	1 (16.7%)	3 (8.3%)	4 (8.0%)	0.474
Eventration	1(16.7%)	1 (2.8%)	2 (4.0%)	0.268
Bowel/Stoma obstruction	0 (0.0%)	1 (2.8%)	1 (2.0%)	0.857
Systemic complications	0 (0.0%)	3 (8.3%)	3 (6.0%)	0.621
Other	0 (0.0%)	4 (11.1%)	4 (8.0%)	0.526
Late	2 (33.3%)	11 (30.6%)	13 (26.0%)	0.615
Stoma prolapse	1 (16.7%)	6 (16.7%)	6 (12.0%)	0.743
Stoma stricture	0 (0.0%)	4 (11.1%)	4 (8.0%)	0.526
Parastomal hernia	1 (16.7%)	2 (5.6%)	3 (6.0%)	0.378
Parastomal ulceration	0 (0.0%)	1 (2.8%)	1 (2.0%)	0.857
Bowel obstruction	0 (0.0%)	1 (2.8%)	1 (2.0%)	0.857
Other	1 (16.7%)	2 (5.6%)	3 (6.0%)	0.378
Presence of early and late complications	1 (16.7%)	2 (5.6%)	3 (6.0%)	0.378
Mortality	0 (0.0%)	17 (47.2%)	17 (34.0%)	0.065
In-hospital mortality	0 (0.0%)	1 (2.8%)	1 (2.0%)	0.857
Related to primary disease and RVF treatment	0 (0.0%)	7 (19.4%)	7 (14.0%)	0.309
Unrelated to primary disease and RVF treatment	0 (0.0%)	10 (27.8%)	10 (20.0%)	0.308

Other early complications: episode of postoperative fever above 38 °C without known underlying cause, decrease in Hg value without signs of active bleeding, urinary bladder lesion. Other late complications: intra-abdominal abscess, stenosis of the rectum, phlegmon of the anterior abdominal wall

### Factors prognostic of treatment outcomes

Univariate analysis (Table 1) found distance from the anal verge >7 cm (*p* = 0.007 OR 18 95%CI 2.2609–14.3062) and creation of loop ileostomy (*p* = 0.08 OR 17 95%CI 1.2818–23.9701) to significantly correlate with a positive outcome, whereas a prolonged course of radiotherapy of over 6 weeks (*p* = 0.047) correlated negatively. In multivariate analysis, only distance from the anal verge remained significant (*p* = 0.031 OR 2.35 95%CI 1.0422–5.2924; model significance *p* = 0.01).

### Quality of life

All living patients, who were examined at the end of the study in 2015, agreed to complete the questionnaires. Among them were 6 patients with repaired fistula and 14 patients with persistent fistula.

Analysis of the *AIS questionnaire* showed a median score of 27 (range 11–39), indicating moderate acceptance of the disease by patients. The group with cured RI-RVF gave higher scores, indicating good acceptance of illness (median 35 vs 27, *p* = 0.0814); however, this was found to be statistically insignificant.

For the *SWLS questionnaire*, the median score from all patients was 22 (range 15–32), indicating the respondents to be generally satisfied with life. No patient was classified as dissatisfied. The obtained scores did not differ statistically between patients with and those without repaired fistula (median 22 vs 21, *p* = 0.5714).

## Discussion

In 2014, nearly 100,000 people received radiation therapy for gynaecological malignancies in the USA [17], at least, 4% of whom will develop radiation-induced rectovaginal fistula. It is estimated that for every 1000 cancer cases in a population, about 52% of patients need radiation therapy as an optimal part of their management and a further 23% of this number will require retreatment [18]. Recent decades have also seen a progressive increase in disease-free survival, increasing the numbers of patients who may develop RVF, long after oncological treatment is finished. The actual numbers of patients with RI-RVF are not easily available since there are no population-based registries concerning RI-RVF worldwide. That is why the scale of the problem may not be properly perceived. Careful analysis of published reports concerning radiotherapy for endometrial and cervical cancers which refer to Polish population in the last decade reveals incidence of RI-RVF in 3% of patients in analysed groups [19, 20]. We ourselves have enrolled 50 patients for the participation in the study from 1725 women who were treated with radiotherapy for stages I–III of cervical and endometrial cancer with the curative intent for the last decade in Regional Oncologic Centre in Lodz. In this case, the incidence is estimated to be about 3% too. It is generally agreed that doses above 70 Gy cause significant and long-lasting injury to the surrounding area [21] [22]. Although numerous factors have been identified increasing the risk of RI-RVF like advanced primary tumour stage and elevated doses of radiotherapy to the rectum, as well as the presence of cardiovascular comorbidities, smoking and surgery or biopsy in the previously irradiated field, the risk of RI-RVF development cannot always be modified by the patient or physician [14, 23–25]. Our findings indicate that RI-RVF develops spontaneously, usually after 2 years following radiotherapy, and is not precipitated by major events such as pelvic floor surgery or trauma.

The current literature affords little attention to the matter of radiation-induced rectovaginal fistulas. Moreover, no reliable clinical trials have yet been published concerning the efficiency of available treatment options [7]. Numbers of papers concerning treatment of RVF of different origins presents a variety of techniques available to use, both from perianal and abdominal approach. Success rates in such operation varied from 25 to 100% and often rise after repeated surgery [10, 12, 14, 26]. Which of them refers to RI-RVF? D’Ambrosio et al. report the results of RVF treatment with transanal endoscopic microsurgery (*TEM*) in a group of 13 patients [27]. The approach led to fistula healing in 12 patients, but the only one not healed had radiation-induced RVF, with two relapses following treatment. After a further unsuccessful treatment of the relapse with TEM and the presence of another relapse, the patient refused another attempt and maintained her ostomy. In 2007, McNevin achieved a healing rate of 96% when using the Martius flap. However, none of the patients had experienced RI-RVF and post-procedural dyspareunia was also observed [6]. In a systematic review of other literatures and his own experience (2008), Wexner reports a success rate of 73.5% (range 33–100%) with 81.8% of stoma closures after gracilis muscle interposition [14]. Complications were present in 29% and this often demanded more than one repair. Authors claimed that such results were harder to achieve in the inflammatory bowel disease and radiation group. Also, the use of plugs and fibrin glue in RI-RVF is not recommended, since short and frequently epithelialized canal of the fistula is not suitable for such modality [10]. In all of the reports, patients with RI-RVF comprised usually not more than 20% of the analysed patients. Among the operations from the abdominal approach that have been proved to be useful in treating RI-RVF, coloanal sleeve anastomosis and the Bricker-Johnston sigmoid colon graft were applied in a larger group of patients, though majority of the reports were published around 1980–1990. Coloanal sleeve anastomosis, first described by Parks and colleagues in 1978, is used now occasionally to treat rectovaginal fistulas [28]. However, in 1986, Cooke and Wellsted achieved healing of the post-radiation fistula in 55 of 59 patients (93%) and Nowacki reported good functional outcomes in 18 of 24 patients with RI-RVF, treated over a 10-year period [9, 29]. Bricker-Johnston sigmoid colon graft [30] and its modification proposed by Steichen [31] was successfully applied in a 61-year-old female with RI-RVF with good functional results but following the three-stage procedure. More recently, Zimmermann described the ultra-deep anterior rectum resection with transperineal and transvaginal omentum reconstruction coupled with protective ileostomy construction that his team used to treat a 37-year-old woman with RI-RVF [32]. Authors of these reports observed that the described techniques are efficient in cases of high fistulas and emphasized the careful selection of patients fit for the procedures. Both of the observations are confirmed in our study. The only factor significant in multivariate analysis was a high location of the fistula that correlates positively with spontaneous RI-RVF healing. Although statistical strength could not be high for the analysed cohort, the fact that high fistula is more prone to heal seems to be obvious. This may be attributed to a number of possible causes. In these patients, the sphincter mechanism is usually not impaired significantly and anastomosis can be made 1–2 cm above the dentate line, giving better functional outcomes and possibility to perform resection and/or interposition of healthy tissue. Recently, the role of colonic bacteria in the upper part of the rectum is postulated as a factor fostering healing. Colonic bacteria are known to produce short-chain fatty acids (SCFAs), which are the main oxidative fuel for the colonic mucosa and have a trophic effect on the rectal mucosa stimulating mucosal blood flow [33]. We observed 11 patients with high fistulas and in 3 patients fistula healed spontaneously. In general, we performed lower anterior resection in two patients. In the remaining cases, the creation of a faecal diversion and occasional simple suturing of the fistula canal was applied. This in turn refers to second issues discussed by authors mentioned above. In nearly 80% of our patients, the presence of adhesions was observed, often accompanied by inflammatory changes in the pelvis. That is why, resections were not safe and even diverting stoma had to be made on the transverse colon instead of the sigmoid colon. One might ask why we refrained from performing the procedures described above and limited ourselves to creation of faecal diversion only. First of all, the creation of diverting stomy gives the immediate relieving of symptoms and enables the damaged area to heal, thus preparing the patient for the potential further intervention. Despite loop colostomy being advantageous in patients with significant comorbidities and severe local inflammation due to low rates of postoperative complications and the relatively small surgical intervention, the complications did appear and were the reason for reoperations in 15% of the patients (Table 3). Although the majority of them were late and related to stoma, it could prompt the idea that these patients may be of higher risk for developing complications. Only one patient died in the hospital, but 41% of all deceased patients in follow up were due to cancer and RI-RVF treatment. For this reason, the authors repeatedly consulted surgeons from well-known colorectal departments abroad, which sustained the decision of not performing more advanced procedures. Not only do current oncological status, patient age, location of the fistula in the rectum, the presence of comorbidities and local tissue status determine the available treatment but also the ability to follow up the postoperative recommendation. Such factors diminished the numbers of patients fitted for surgery, which explain why only 20% of the patients mentioned in new reports are those with RI-RVF. The incidence of RI-RVF remains similar among Western countries, but patients who were not qualified for reconstructive procedures are not counted in the papers. Authors of the study performed various procedures including Martius flap and gracilis muscle interposition with good functional outcomes but in cases of RVF after delivery or trauma. This is a reason why loop colostomy comprised the majority in the analysed cohort. On the other hand, faecal diversion alone rarely leads to spontaneous closure of the fistula [8]. Loop colostomy was treatment of choice in the current study, also to give the patient the opportunity for easier stoma reversal in the future, which was achieved in one patient. Patients underwent essential examinations (computed tomography and colonoscopy) to rule of recurrence, confirm the closure of RI-RVF and check the large bowel for the presence of strictures or stenosis. Finally, the patients were assessed by anaesthesiologists for their fitness for operation, referring patient to specialist (cardiologist, endocrinologist) when necessary. The risk and benefits of the procedure were also widely discussed. The procedure was performed with open access but utilizing the stoma opening and postoperative course was uneventful. However, five patients did not want to undergo another operation. All these raise the question of whether any pretreatment indicators of outcome can be found. Patient age and general health status (expressed in ASA and Charlson score, BMI and smoking status), though possibly indirectly contributing to tissue condition, were not significant predictors of RI-RVF healing. On the other hand, these variables are usually taken into account when qualifying the patient for a particular operation; they therefore may well be related to perioperative risk and the development of complications rather than to directly influence the outcomes of RI-RVF repair. In fact, the main advantage of the perianal procedures is the lack of any significant perioperative morbidity, whereas the placement of viable tissue is usually possible during procedures from the abdominal approach. Still, the main contraindication for the abdominal operations is the significant risk of perioperative complications. The laparoscopic approach may be an optimal solution in this regard; however, it has only been used in a few case studies [13, 34]. Further studies involving larger numbers are warranted, especially in groups with RI-RVF, to provide clear evidence that laparoscopic approaches are safe and beneficial in this group of patients.

## Limitations and strengths

This is so far the largest study analysing the surgical outcomes of RI-RVF treatment and was based on careful data acquisition and a long follow up. However, due to the general health status of the patients and the decision of the surgeon, only faecal diversion and standard resection were instigated in treatment, either with or without coloanal anastomosis, which excluded the varieties of other techniques described above. Although such operations are performed in our department for RVF of different aetiologies, they were not recommended for that particular group. Hence, this study serves as a reminder that most approaches to treating RI-RVF may be used only in a limited number of patients, where local tissue viability and a satisfactory general condition allow their use. However, in a number of patients, a fecal diversion will improve symptoms and their quality of life to the point that they do not require any further intervention even though the underlying problem is not repaired.
